# Contemporary review of IgA nephropathy

**DOI:** 10.3389/fimmu.2024.1436923

**Published:** 2024-08-12

**Authors:** Edward J. Filippone, Rakesh Gulati, John L. Farber

**Affiliations:** ^1^ Division of Nephrology, Department of Medicine, Sidney Kimmel Medical College at Thomas Jefferson University, Philadelphia, PA, United States; ^2^ Department of Pathology, Sidney Kimmel Medical College at Thomas Jefferson University, Philadelphia, PA, United States

**Keywords:** IgA nephropathy, glomerulonephritis, hematuria, proteinuria, complement inhibition, thrombotic microangiopathy, podocytopathy, immunossuppression

## Abstract

IgA nephropathy (IgAN) is considered the most common primary glomerulonephritis worldwide with a predilection for Asian-Pacific populations and relative rarity in those of African descent. Perhaps 20%–50% of patients progress to kidney failure. The pathogenesis is incompletely understood. Mesangial deposition of immune complexes containing galactose-deficient IgA1 complexed with anti-glycan IgG or IgA antibodies results in mesangial cell activation and proliferation, inflammatory cell recruitment, complement activation, and podocyte damage. Diagnosis requires a biopsy interpreted by the Oxford criteria. Additional pathologic features include podocytopathy, thrombotic microangiopathy, and C4d staining. Biomarkers predicting adverse outcomes include proteinuria, reduced GFR, hypertension, and pathology. Acceptable surrogate endpoints for therapeutic trials include ongoing proteinuria and rate of eGFR decline. The significance of persisting hematuria remains uncertain. The mainstay of therapy is supportive, consisting of lifestyle modifications, renin–angiotensin inhibition (if hypertensive or proteinuric), sodium-glucose-transporter 2 inhibition (if GFR reduced or proteinuric), and endothelin-receptor antagonism (if proteinuric). Immunosuppression should be considered for those at high risk after maximal supportive care. Corticosteroids are controversial with the most positive results observed in Chinese. They carry a high risk of serious side effects. Similarly, mycophenolate may be most effective in Chinese. Other immunosuppressants are of uncertain benefit. Tonsillectomy appears efficacious in Japanese. Active areas of investigation include B-cell inhibition with agents targeting the survival factors BAFF and APRIL and complement inhibition with agents targeting the alternate pathway (Factors B and D), the lectin pathway (MASP-2), and the common pathway (C3 and C5). Hopefully soon, the *who* and the *how* of immunosuppression will be clarified, and kidney failure can be forestalled.

## Introduction

Initially described by Berger and Hinglais in 1968 (1), IgA nephropathy (IgAN) is characterized by the biopsy finding of dominant or codominant deposition of IgA-containing immune complexes with a variable inflammatory and/or sclerosing response (2). The clinical presentation is variable, ranging from recurrent bouts of macroscopic hematuria, following either an upper respiratory infection (synpharyngitic nephritis) or gastrointestinal infection, urinary sediment abnormalities (microscopic hematuria and/or proteinuria), to more serious presentations (frank nephrotic syndrome or rapidly progressive glomerulonephritis).

IgAN is typically referred to as the most common form of primary glomerulonephritis worldwide. However, variability exists among different populations with especially high relative frequencies in the Asia-Pacific region (China and Japan) and most Western European counties (up to 50% or more of biopsies with primary glomerulonephritis). IgAN is found less frequently in other areas, e.g., Africa and India (3). Such variability may result from multiple factors, including genetic susceptibility, the availability of immunofluorescence microscopy, differences in mass urinary screening programs, nephrology referral practices, and the willingness of nephrologists to biopsy cases with only mild urinary sediment abnormalities (3).

Although initially considered relatively benign, perhaps 25%–50% of patients with primary IgAN develop end-stage kidney disease (ESKD) if followed for 20–30 years (4). Pitcher et al. followed 2,439 IgAN patients (2,299 adults) referred to the UK National Registry of Rare Kidney Diseases (RaDaR) with either >0.5 g/day of proteinuria or eGFR <60 (all eGFR herein expressed in ml/min/1.73 m^2^) and found median kidney survival of 11.4 years with a mean age of kidney failure/death of 48 years (5). Given age at onset and baseline eGFR, it was estimated that almost all patients could progress to ESKD in their expected lifetimes, unless the average decline in eGFR was <1/year. Mortality is increased with IgAN. A Korean study of 1,364 IgAN patients revealed overall mortality to be 43% greater than the general population, but significantly so only in men (6). A nationwide Swedish cohort study estimated a reduced life expectancy of 6 years (7).

Demographic features affect the clinical presentation and course of IgAN. Originally thought to affect predominantly young adults in the second to fourth decade, the age of diagnosis has been increasing. In the RaDaR study, the median age at diagnosis in adults increased from 40 years in 2013 to 45 years in 2020 (5). The average age of presentation among 2,961 Spanish patients increased from 37.6 years in 1994–1997 to 44.9 years in 2010–2013 with approximately 10% >65 years old (8). Whereas younger patients were more likely to present with gross hematuria and urinary sediment abnormalities, the elderly were more likely to present with reduced eGFR, hypertension, acute kidney injury (AKI), and higher levels of proteinuria.

Racial and ethnic factors play a role in the development and progression of IgAN. Pacific-Asian populations show a greater prevalence than European–Caucasians, have more severe clinical presentations, more active lesions on biopsy, and have an increased rate of progression to ESKD (9, 10). IgAN is much less common in African Americans but does occur (11). Furthermore, racial/ethnic differences in response to therapy are reported with Chinese apparently more responsive to corticosteroids than Caucasians and Japanese more responsive to tonsillectomy (*vide infra*).

The genetic predisposition to IgAN has been extensively investigated (12). Kiryluk et al. performed genome-wide association study (GWAS) and meta-analysis of 17 international cohorts and identified 30 risk loci, which explained 11% of disease risk (13). Asian populations are enriched in susceptibility alleles (14). Additionally, epigenetic factors, such as altered DNA methylation (15) and interference from micro RNAs (16, 17), may contribute to risk.

The current understanding of the pathogenesis of IgAN is thought to involve four sequential hits (18). Elevated serum levels of primarily polymeric IgA1 with galactose-deficient hinge regions (Gd-IgA1) represent Hit 1 but are clearly not sufficient, as unaffected, healthy, first-degree relatives of patients with IgAN can have elevated levels. Gd-IgA1 forms polymers by self-aggregating. Hit 2 involves production of anti-Gd-IgA1 autoantibodies, predominantly IgG (but also IgA1). Hit 3 involves formation in the serum of immune complexes of polymeric IgA1 and anti-Gd-IgA1 of sufficient size to escape hepatic clearance and deposit in the glomerular mesangium. Finally, mesangial cells are activated, proliferate, recruit inflammatory cells, and secrete cytokines affecting podocyte structure and function (Hit 4). Importantly, both Gd-IgA1 and anti-GdIg-A1 antibodies are found in normal serum, albeit at lower levels than in IgAN patients (19). The naturally occurring anti-Gd-IgA1 may have been initially induced by various microorganisms (viruses and bacteria) that express the N-acetylgalactosamine residue characteristically overexposed by galactose deficiency in the Gd-IgA1 of IgAN patients (20).

## Biomarkers and surrogate endpoints in IgAN

A biomarker in IgAN is a clinical or laboratory feature that predicts future development of a hard adverse outcome (ESKD, cardiovascular events, death). A valid surrogate endpoint is a biomarker whose alteration with a given therapy predicts a subsequent reduction in future hard endpoints with that therapy. Given the slowly progressive course of IgAN, hard clinical endpoints would not be feasible for therapeutic trials, and surrogate endpoints are needed. Surrogate endpoints may be validated or “reasonably likely.” A therapeutic benefit on a “reasonably likely” surrogate endpoint allows for accelerated FDA approval, although post-marketing trials are required to verify benefit. This is most germane when considering potentially toxic immunosuppressive therapy.

No biomarker is acceptable to diagnose IgAN, and biopsy is required. The most established biomarkers for predicting progression of IgAN at presentation include proteinuria, reduced eGFR, and hypertension clinically (21, 22) and the Oxford criteria pathologically. Potential clinical biomarkers requiring verification include genetic features, hematuria, levels of Gd-IgA1 and anti-Gd-IgA1 antibodies, and the IgA/C3 ratio. Additional pathologic findings indicating progression include thrombotic microangiopathy and positive C4d staining. Proteinuria, declining eGFR, persisting hematuria, and histologic changes on repeat biopsy are potential surrogate endpoints for clinical trials.

The level of Gd-IgA1 is a potential diagnostic tool and biomarker. Galactose-deficient IgA1 can be determined by various methods, including Western blotting, mass spectrometry, and liquid chromatography, but these are not readily available. Several lectins can bind to the unglycosylated N-acetylgalactosamine on Gd-IgA1, including lectins derived from the snail *Helix aspersa* (HAA) and the plant *Vicia velosa.* Moldoveanu et al. developed an HAA-based quantitative enzyme-linked immunosorbent assay (ELISA) for determining serum levels of Gd-IgA1 (23). Yasutake et al. developed an ELISA based on a rat monoclonal antibody (KM55) against human Gd-IgA1 correlating with the HAA-based assay (24).

As noted above, Gd-IgA1 is normally present in serum. Moldoveanu et al. found the 90th percentile using the HAA ELISA to be 1,076 U/ml in 150 healthy Caucasian adults (23). Sun et al. did a systematic review and meta-analysis of studies using lectin-based assays and showed that patients with IgAN have significantly higher mean levels Gd-IgA1 compared to normal but not different than first-degree relatives of IgAN patients or patients with IgA vasculitis. Levels in IgAN patients did not correlate with disease severity (25). Wada et al. used KM55 and found significantly higher serum levels of Gd-IgA1 in IgAN patients versus patients with lupus nephritis, ANCA-vasculitis, or minimal change disease, but similar levels to patients with IgA-vasculitis (26).

Similarly, anti-Gd-IgA1 antibodies are found in normal serum but at lower levels than in IgAN patients. Yanagawa et al. found that 41% of Japanese IgAN patients had serum levels of Gd-IgA1 above the 90th percentile of normal, and 91% had anti-Gd-IgA1 IgG antibodies above the 90th percentile of normal, as did up to 25% of patients with other kidney diseases, especially immune-mediated diseases such as lupus nephritis (27). The level of anti-Gd-IgA1 IgG performed well in separating IgAN patients from normal controls but less so compared to other immune-mediated kidney diseases.

Vaz de Castro et al. performed a systematic review and meta-analysis of observational studies and clinical trials where Gd-IgA1 levels were measured (29 studies involving 8,159 participants) (28). Levels were not significantly associated with hypertension, hematuria, or proteinuria. Results relating Gd-IgA1 levels to chronic kidney disease (CKD) stage or progression to ESKD were inconsistent. Currently, measurement of serum levels of Gd-IgA1 and/or corresponding antibodies are not sufficiently sensitive or specific to preclude biopsy and are not recommended as standard biomarkers of progressive disease.

Several studies assessed the serum IgA level/C3 ratio as a biomarker for diagnosis, grading severity, and/or determining prognosis. Tomino et al. found that the ratio could distinguish IgAN from other glomerulopathies in Japanese patients (29). Kawasaki et al. found that an elevated IgA/C3 ratio combined with more intense glomerular C3 staining predicted greater proteinuria, hematuria, and creatinine at follow-up, as well as more severe pathology scores on a second biopsy in Japanese pediatric IgAN patients (30). Mizerska-Wasiak et al. found more severe pathologic changes associated with IgA/C3 in pediatric Polish IgAN patients (31). By studying 1,219 Chinese adult patients, Chen et al. looked specifically at Gd-IgA1 and found a linear relationship between the serum Gd-IgA1/C3 ratio and kidney disease progression (32). More data are needed before general recommendations can be made regarding these ratios. Multiple other biomarkers in serum and urine have been proposed, but none are sufficiently validated for routine use (33, 34).

## Proteinuria

Proteinuria is the best studied baseline biomarker and surrogate endpoint. Reich et al. followed 542 patients from the Toronto Glomerulonephritis Registry and found that 30% reached ESKD; follow-up proteinuria was the most important predictor by multivariable analysis (35). The rate of eGFR decline was 25-fold faster if the average proteinuria was >3 g/day versus <1 g/day. Reduction from >3 to <1 g/day produced a similar course to having <1 g/day throughout. Inker et al. performed an individual patient level meta-analysis of 11 randomized trials (830 patients) evaluating four interventions and found that decline in proteinuria at 9 months was significantly associated with reduced risk of doubling of serum creatinine, ESKD, or death [hazard ratio (HR) 0.40, 95% confidence interval (CI) 0.32–0.48] (36).

The Kidney Health Initiative extended this analysis to include 13 randomized controlled trials (RCTs) and 7 observational cohorts restricted to patients with IgAN (37) confirming the association of proteinuria reduction with hard clinical outcomes (doubling of serum creatinine, ESKD, or death). Proteinuria reduction is now considered a “reasonably likely” surrogate endpoint (see https://www.fda.gov/downloads/Drugs/Guidances/UCM358301.pdf) (37) that allowed for accelerated FDA approval of budesonide and sparsentan (*vide infra*).

Although Kidney Disease Improving Global Outcomes (KDIGO) considers proteinuria >0.75–1 g/day as high risk and reduction to <1 g/day as a reasonable target (38), patients with lower levels clearly may still progress. In the RaDaR study, approximately 30% of patients with time-averaged proteinuria <0.88 g/g creatinine (approximately <1 g/day) reached ESKD by 10 years, as did approximately 20% with <0.44 g/g (approximately <0.5 g/day) (5).

The nephrotic syndrome (NS), defined as nephrotic range proteinuria (NRP) with hypoalbuminemia, is found in approximately 10%–15% of primary IgAN patients (39, 40). The prognosis for IgAN is worse with NS. In one study, 24% of NS patients had doubling of serum creatinine over 45 months versus 7% of 885 patients without NS (40), and achieving remission significantly improved survival from that endpoint. Interestingly, 25% of nephrotic patients underwent spontaneous remission without relapse. In another study of 24 nephrotic IgAN patients, 5 had spontaneous remission, again with no relapse. In contrast, Huang et al. studied 1,413 Chinese IgAN patients and found no significant difference in the doubling of serum creatinine or ESKD in the 57 with NS (11).

Four studies of Chinese IgAN patients compared the prognosis of NS to NRP with normoalbuminemia (41–44). Three of these specifically excluded cases of IgAN with biopsies otherwise consistent with minimal change disease (MCD), the so-called MCD–IgAN (*vide infra*) (41, 42, 44). Li et al. compared 59 NS patients to 57 patients with NRP and found greater endocapillary inflammation and crescents in the former, but greater segmental sclerosis/adhesions to Bowman’s capsule in the latter. However, there was no significant difference in long-term outcome (41).

Chen et al. studied 119 patients with >2 g/day of proteinuria that were divided into 33 with NS, 34 with NRP but normoalbuminuria, and 52 controls with >2 but <3.5 g/day of proteinuria. Patients with NS had greater hematuria, more likely endocapillary inflammation, and more severe foot-process effacement (42). Survival free from a combined endpoint of doubling of serum creatinine and 50% reduction of eGFR or ESKD was surprisingly significantly better with NS versus NRP, and both were significantly worse than the controls.

Jiang et al. compared 85 patients with NS to 85 patients with only NRP and found the former had baseline higher eGFR, more severe hematuria, more severe foot process effacement, lower serum C3 levels, and lower body mass index (BMI) despite similar pathologic scores (44). Those with NS had double the risk of ≥30% decline in eGFR or ESKD on follow-up by Cox regression, although the rate of decline in eGFR was similar.

These three studies that excluded MCD–IgAN provide no clear evidence of worse outcome with NS versus NRP. The study not excluding MCD–IgAN compared 111 patients with NS to 111 with NRP and found no difference in composite outcome unless patients with complete remission were excluded in which case NS portended a worse outcome (43).

## eGFR

Reduced baseline eGFR is a strong biomarker for an unfavorable outcome in IgAN. The rate of decline in eGFR has been extensively studied as a potential surrogate across a spectrum of kidney diseases. Inker et al. performed a meta-analysis of 47 RCTs assessing 12 interventions in over 60,000 patients and found a significant relationship between slope difference and hard clinical outcomes (45). For example, a difference in slope in the range of 0.5–1.0 ml/min/1.73 m^2^ from baseline to 3 years would have >97% chance of reducing adverse outcomes. Shorter time frames (e.g., 1 year) produce less certainty due to generally opposing short-term acute effects of many therapies. Similar results were obtained when analyzing observational data (46).

When analyzing 1-year data from 13 RCTs of IgAN therapy, Lafayette et al. found that both 1-year slope of eGFR decline and 1-year reduction of proteinuria were significantly associated with a composite end-point (eGFR reduction, serum creatinine doubling, ESKD, and/or death) (47). However, by multivariable analysis, only eGFR slope remained highly significantly associated with the composite endpoint, but proteinuria change did not.

## Pathology: biomarker and surrogate?

The Oxford classification of IgAN (see **Table 1**), was published in 2009 to both standardize the pathologic description of IgAN and, importantly, to provide prognostic information (49, 50). Measures of both activity (mesangial hypercellularity, M, and endocapillary hypercellularity, E) and chronicity (segmental sclerosis, S, and tubular atrophy/interstitial fibrosis, T) were included, termed the MEST score. The prognostic ability has been substantiated multiple times [19 studies reviewed in (48)] including in both Asian (51) and European populations (52, 53). It was estimated that adding the MEST score to clinical features at biopsy would provide an equivalent predictive ability to 2 years of following clinical variables (53).

**Table 1:** Mest-C score (48).

	Description	Criteria
**M**	Mesangial proliferation: ≥4 mesangial cells/mesangial area	M0: <50% glomeruli involved M1: >50% glomeruli involved
**E**	Endocapillary hypercellularity: Hypercellularity within a glomerular capillary loop causing luminal narrowing	E0: absent E1: present in at least 1 glomerulus
**S**	Segmental glomerulosclerosis: Any amount of tuft sclerotic other than the complete tuft or any adhesion to Bowman’s capsule	S0: absent S1: present in at least 1 glomerulus*
**T**	Tubular atrophy/interstitial fibrosis: Percentage of cortical area involved by either tubular atrophy or interstitial fibrosis, whichever is greater	T0: <25% T1: 26%–50% T2: >50%
**C**	Crescents: Cellular or fibrocellular crescents	C0: absent C1: present in at least 1 glomerulus C2: present in >25% of glomeruli

Total number of glomeruli should be noted.

Note the number of glomeruli with endocapillary hypercellularity, necrosis, cellular/fibrocellular crescents, segmental glomerulosclerosis, and global glomerulosclerosis.

*Note the presence or absence of podocyte hypertrophy and a glomerular tip lesion in S1 biopsies.

Unsurprisingly, the T lesion has been most consistent in independently predicting adverse renal outcomes [in 14 of 19 studies by multivariable analysis (48)] since the endpoint in these studies was often ESKD. The E lesion was not predictive in most studies, but this was influenced by immunosuppression, as E1 biopsies were more likely to be immunosuppressed. The E lesion was predictive when analyzing only un-immunosuppressed patients suggesting it may be responsive to therapy. The S lesion may arise from healed necrotic or inflammatory lesions or be a result of primary podocyte damage. At least one study showed that either podocyte hypertrophy or the tip lesion was associated with an adverse outcome that was improved with immunosuppression (54). The presence or absence of both should be noted in the pathology report.

Crescents were not found to significantly predict adverse outcomes in the original Oxford classification presumably because rapidly progressive cases with eGFR <30 were excluded. The median number with crescents was only 9% also limiting predictive ability (49). This issue was addressed by Katafuchi et al. in 702 Japanese patients, including 254 with low-level proteinuria, 32 with eGFR <30, and 416 who met original Oxford inclusion criteria (55). Crescents were significantly associated with kidney survival overall and in the 286 not meeting Oxford criteria, but not in the 416 meeting those criteria. The predictive ability of crescents was validated in two pediatric studies (56, 57).

Haas et al. retrospectively analyzed 3,096 patients from four cohorts and found that crescents significantly predicted >50% decline in eGFR or ESKD overall, although only in patients not immunosuppressed when considered separately. However, having crescents in >25% of glomeruli was predictive regardless of immunosuppression. In 2016, crescent scores were added (now the MEST-C score) (48).

Additional pathologic changes not considered in the MEST-C score have a significant impact on the clinical presentation, prognosis, and response to therapy, most notably podocytpathy, thrombotic microangiopathy, and positive C4d staining (see **Table 2**).

**Table 2:** Beyond MEST-C: additional pathological considerations.

Category	Pathology	Significance	Comment
**Podocytopathy**			
MCD–IgAN	Diffuse foot process effacement with either no changes by light or mild mesangial hypercellularity Deposits by IF and EM limited to mesangium	Presents with florid nephrotic syndrome Very responsive to steroid therapy	May represent adult MCD with lanthanic IgA deposits May also represent IgAN variant with cytokine-derived podocyte injury
S1	Defined as segmental sclerosis in ≥1 glomerulus or ≥1 adhesion Note the presence or absence of podocyte hypertrophy and/or tip lesion	Podocyte hypertrophy and tip lesions correlate with greater proteinuria and more rapid decline in eGFR May be more responsive to therapy	Suggests subdivision of S1 based on defining feature: Only adhesions Segmental sclerosis ± adhesions ± PH Remains to be determined if applying full Columbia classification of FSGS is warranted
**Thrombotic microangiopathy**	Acute: arteriolar or glomerular thrombi, fragmented red cells, fibrinoid necrosis Chronic: arteriolar intimal hyperplasia (“onion skinning”), mesangiolysis, GBM double contour EM: widened subendothelial space	Associated with a more severe clinical picture in multiple studies in various populations Associated with malignant hypertension Associated with concurrent podocytopathy	Found in a variable percentage of biopsies Should be considered in future Oxford iterations May occur with frank normotension May foster podocytopathy
**C4d**	May be positive in glomeruli and/or arterioles	Associated with a more severe clinical picture and worse outcome	Found in about one-third of biopsies Supports lectin pathway involvement May be a biomarker to guide anti-lectin therapy

EM, electron microscopy; FSGS, focal segmental glomerulosclerosis; IF, immunofluorescence microscopy; MCD, minimal change disease; PH, podocyte hypertrophy.

## Podocytopathy in IgA nephropathy

Proteinuria is the feature most indicative of podocyte injury or loss. Podocyte foot process effacement is well described in primary IgAN, the degree of which directly correlates with the amount of proteinuria (58, 59). Podocytopenia has been found in IgAN with an apparent threshold of 250 podocytes per glomerulus below which proteinuria increases and GFR decreases (60). In pediatric IgAN, cumulative urinary podocyte excretion over time significantly correlated with progressive glomerulosclerosis on serial biopsies (61).

The underlying pathology and associated clinical course of NS in IgAN patients is quite variable. Early reports centered on biopsies of MCD–IgAN and were characterized by either no light microscopic changes or only mild mesangial proliferation. Both the IgA deposits by immunofluorescence and the electron dense deposits by electron microcopy (EM) were restricted to the mesangium (62–69). Clinically, these patients typically had florid NS, normotension, preserved eGFR, and displayed marked steroid sensitivity with little tendency to progress. MCD–IgAN is not rare. For example, Han et al. reported a series of 1,336 Chinese IgAN patients that included 171 with MCD–IgAN and 171 others also with NS and pathology other than minimal glomerular abnormalities/diffuse foot process effacement (70). In the series of 57 Chinese IgAN patients of Huang et al. noted above, 13 (22.8%) had MCD–IgAN (11).

The pathophysiology of MCD–IgAN is uncertain. Some posit that it simply represents the coexistence of two glomerulopathies, MCD with a mild IgAN (67). It is also possible that these cases are simply MCD with lanthanic IgA deposits of otherwise no consequence. Asymptomatic IgA deposits are not rare in the general population. Waldherr et al. found IgA deposits in 12 of 250 consecutive autopsy cases without known kidney disease; only one had C3 deposits (71). Suzuki et al. found latent IgA deposits in 82 (16%) of 510 Japanese allografts at the time of implantation along with C3 in 16/82. These 16 had a significantly higher incidence of mesangial proliferative GN (50%) and greater macrophage infiltration but only a minority had increased urinary erythrocytes (72). Similarly, Sofue et al. found latent IgA deposits in 20 of 68 consecutive Japanese live-kidney donors of which 14 had mesangial expansion. There was no significant association of IgA deposits (± mesangial expansion) with allograft survival or function, and a repeat biopsy at 1 year showed that 14/20 no longer had IgA deposits (73). These data support the idea that some MCD–IgAN patients simply have MCD that responds to treatment as such, and that the IgA deposits are latent and otherwise of no consequence.

Additionally, Guo et al. compared 51 MCD–IgAN Chinese patients to both 51 consecutive IgAN patients without MCD and healthy participants as controls. Gd-IgA1 levels were lower in MCD–IgAN than in non-MCD–IgAN cases but higher than in controls, with only rare mesangial deposition of Gd-IgA1 in the MCD–IgAN patients (7/51, 13.7%) compared to non-MCD–IgAN patients (42/51, 82.4%).

Alternatively, MCD–IgAN may represent a primary IgAN podocytopathy. Given that the primary site of immune complex deposition and inflammation is the mesangium in IgAN, the mechanism of podocyte injury requires clarification. Potential mechanisms include toxicity of Gd-IgA1 aggregates directly, mesangial cell production of cytokines injurious to podocytes, or toxicity from C5b-9 generated in the mesangium. Wang et al. demonstrated reduced *in vitro* nephrin expression in podocytes exposed to aggregated IgA1 from IgAN patients and less so with IgA1 from normals, a finding that suggests a direct podocyte effect of abnormal IgA1 (74). Others found neither binding of IgA1 to podocytes nor podocyte expression of mRNA of known IgA receptors, and IgA1 from patients did not stimulate podocyte production of cytokines (75). However, cytokines produced from exposing mesangial cells *in vitro* to IgAN sera resulted in production of cytokines capable of directly activating podocytes (74–78). Candidate cytokines included TNF-α (75), TGF-β (76), angiotensin II (74, 77), and PAF (78).

Other patients with variable degrees of proteinuria, including NRP with or without NS, have more significant inflammatory and/or sclerotic glomerular lesions. Pathological findings may include necrosis, E1, C1/2, and S1 MEST-C scores, as well as varying types of focal and segmental glomerulosclerosis (FSGS), as defined in the Columbia classification (79). The clinical picture may include hypertension, variable proteinuria (up to and including NRP and NS), and reduced eGFR that worsens over time.

The Oxford S1 lesion requires special consideration and is found most frequently with sub-nephrotic range proteinuria. The original Oxford definition of S1 required at least one glomerulus with any degree of segmental sclerosis or the presence of a capsular adhesion. S1 defined as such correlated with clinical severity at diagnosis (blood pressure, eGFR, and proteinuria), as well as rate of decline of kidney function (49). Validation of S1 in the VALIGA cohort of patients from 55 European centers concurred (52). Segmental sclerosis in IgAN may develop by one of three mechanisms: healed segmental inflammatory or necrotic lesions, hyperfiltration from nephron reduction (typically associated with glomerulomegaly and perihilar sclerosis), or as a primary podocytopathic injury as outlined above for MCD–IgAN (54).

Hill et al. found that 92 of 128 French IgAN patients (72%) had capsular adhesions, including 53 with no underlying glomerular lesions, although there was loss of mature podocyte markers (synaptopodin, nephrin, VEGF) surrounding the adhesions. In comparison, 100% of primary FSGS patients, a well-known primary podocytopathy, had adhesions, but only 8% of 100 lupus patients, an immune-complex glomerulopathy like IgAN, had any (80). Applying the Columbia classification of FSGS to the 128 biopsies, 11 had collapsing FSGS, 27 the cellular variant, 4 tip lesions, 7 perihilar, 52 not otherwise specified (NOS), 11 non-definable as FSGS, and 16 had no lesions (81). Those with FSGS lesions associated with underlying proliferative lesions (M1, E1, or necrosis) did the worst long term, but even those with pure FSGS lesions without associated glomerular inflammatory lesions fared worse than those without FSGS.

To determine if subclassification of S1 is warranted, Bellur et al. reanalyzed the original Oxford cohort with available slides and found that 137/202 qualified as S1, of which 38% had podocyte hypertrophy (PH), 7% tip lesions, 3% perihilar sclerosis, and 2% endocapillary foam cells (54). PH and tip lesions were significantly associated with initial proteinuria and demonstrated significantly improved survival free from ≥50% decline in eGFR or ESKD when immunosuppressed. The 2016 Oxford update recommended not subclassifying S1, however, but rather simply specifying whether PH or tip lesions are present (48).

Bellur et al. attempted to validate S1 subclassification by reanalyzing the VALIGA cohort of 1,147 European patients regarding the association of specific podocyte lesions with proteinuria, outcome, and response to immunosuppression (82). They found that 69% satisfied S1 criteria, and the 796 were divided into 502 with any segmental sclerosis (NOS) as a qualifying criterion and 294 only having capsular adhesions with or without PH. The NOS+ group was further divided into those also having adhesions with or without PH to yield four groups: S1 without NOS sclerosis (adhesions only by definition), S1 with NOS but without adhesions or PH, NOS with adhesions without PH, and NOS with both adhesions and PH. They showed progressively worse proteinuria and kidney survival across the four groups. A benefit to immunosuppression was only found in the latter two groups. They advocate subclassifying S1 into active lesions more likely to respond to immunosuppression, i.e., those with adhesions or PH.

## Thrombotic microangiopathy in IgAN

Thrombotic microangiopathy (TMA) of the kidney is defined pathologically as acute or chronic. The former includes cell swelling/subintimal edema, thrombus-occluding arterioles or glomerular capillaries with or without fragmented red cells, and fibrinoid necrosis of vessel walls. Chronic changes include arterial intimal proliferation (“onion skinning”), mesangiolysis, and/or double contours of the glomerular basement membrane by light microscopy, with glomerular capillary basement membrane duplication on EM. There may or may not be an associated microangiopathic hemolytic anemia (MAHA), characterized by anemia, schistocytes, thrombocytopenia, absent haptoglobin, and elevated LDH. Clinically, patients may have various diagnoses based on associated features, including thrombotic thrombocytopenic purpura, hemolytic uremic syndrome (HUS), antiphospholipid antibody syndrome, malignant hypertension, scleroderma renal crisis, radiation nephritis, infections, or drug/medication reactions. Although reported in IgAN, TMA is not considered in the Oxford classification.

A well-described cause of TMA, malignant hypertension, may develop in patients with IgAN with reported incidences ranging from 0.5% of Chinese patients to 15% of Spanish patients with IgAN. Chen et al. compared 45 cases of malignant hypertension complicating IgAN (1.2% of all IgAN cases) to 26 cases of malignant hypertension complicating primary hypertension. They found the IgAN patients had more severe proteinuria and hematuria, lower serum creatinine, and better kidney survival; however, kidney survival was not different when initial creatinine was considered (83). Chang et al. reviewed pathology reports and identified 10 cases of TMA out of 435 cases of IgAN; 6 had malignant hypertension and 3 others severe hypertension (84).

El Karoui et al. studied 128 French patients with IgAN and found that 63 (53%) had pathologic TMA, including 8 with evidence of MAHA (85). Only 18 had malignant hypertension (100% of which had TMA), and 47 had uncontrolled hypertension (66% had TMA), but 63 were normotensive, including 19 on no antihypertensive medications (16% had TMA). No difference was found between those with and without TMA in terms of antiphospholipid antibodies or complement gene mutations, but proteinuria in those with TMA was significantly higher (only 5% had <0.5 g/24 h), as were glomerular lesions and Oxford scores. Notably, 16% of TMA cases had eGFR > 60. Hence, TMA can be found in IgAN with normotension and preserved renal function. Prognosis was worse with TMA overall, but significantly so only in those with MAHA.

Cai et al. evaluated 944 Chinese IgAN patients and found that 194 (20.6%) had arterial/arteriolar TMA lesions compared to 0 of 60 with other glomerulopathies, and 69 of the 194 (37.3%) had glomerular subendothelial changes by EM (86). Patients with TMA had higher blood pressure and proteinuria, lower eGFR, and higher MEST-C scores. Similar to El Karoui et al., only 9.8% had malignant hypertension and 33% did not have hypertension. Seventy-five of 194 (38.7%) with arterial/arteriolar TMA progressed to ≥50% decline in eGFR, ESKD, or death versus 29 of 313 (9.3%) with arterial intimal fibrosis (arteriosclerosis) or arteriolar hyalinosis (arteriolosclerosis) alone versus 54 of 437 (12.4%) with no vascular lesions. By multivariable analysis, TMA conferred a 1.95 hazard ratio for the composite outcome.

Neves et al. found TMA in 21 of 118 Brazilian IgAN patients (17.8%), and TMA patients had more frequent hypertension and hematuria, lower eGFR, a greater E1 MEST-C score, and a significantly greater risk of ESKD. Similarly, Puapatanakul et al. found that 21 of 166 Thai patients (13%) with IgAN had TMA with significant associations with hypertension (81% versus 57% without TMA) and malignant hypertension (38% versus 6%), as well as lower eGFR, higher proteinuria, and a higher T2 MEST-C score. TMA was an independent predictor of ESKD.

In the report of FSGS lesions in IgAN patients mentioned above (81), TMA lesions were found in 6/7 with perihilar FSGS, 19/27 with the cellular variant, 29/52 with FSGS NOS, and 10/11 with collapse. In contrast, only 2/16 with no lesions and 2/11 with lesions not definable as FSGS had TMA. These findings suggest a link between TMA and progressive podocytopathy in IgAN.

The original Oxford classification, and subsequently others (86), did not find a significant association between arteriolar hyalinosis and/or arterial intimal fibrosis with outcome. Thus, vascular changes are not included in MEST-C scoring. In contrast, Nasri found that only 2 of 136 Pakistani IgAN patients had a TMA but did find a significant correlation of both arteriolar hyalinosis and arterial intimal fibrosis with serum creatinine and between arteriolar hyalinosis and amount of proteinuria (87).

Importantly, the lesions of TMA were not considered in the Oxford analyses. Given the significantly positive associations of such lesions with outcome noted above, TMA should be considered in future Oxford iterations.

## C4d

C4d staining by immunofluorescence or immunohistochemistry reflects complement activation by either the classic or lectin pathways. In IgAN, lectin pathway activation is presumed to be the cause of positive staining, since mannose-binding lectin (MBL), L-ficolin, and MBL-associated serine proteases (MASPs) are found (88), whereas C1q is infrequently positive. Multiple studies assessed glomerular C4d staining as a biomarker for adverse outcomes. Jiang et al. performed a systematic review and meta-analysis and identified 12 studies (cohort and cross-sectional) with 1,251 patients and found a 34% prevalence of glomerular C4d staining. Significant heterogeneity existed, and definitions of positivity ranged from any non-sclerotic glomeruli to >75% positive. Asian populations had numerically higher positivity rates than Caucasian populations (46% versus 30%, p = NS). Patients with C4d deposition had lower eGFR, higher urinary protein, more hypertension, and higher Oxford scores. Glomerular C4d staining was independently associated with adverse kidney outcomes including ESKD.

Faria et al. assessed the prevalence and significance of arteriolar and glomerular C4d staining in 126 Portuguese adults and found arteriolar staining (defined as >25% of arterioles) in 17% that was associated with blood pressure, arterial intimal fibrosis, and chronic microangiopathy (89). Such staining remained a significant biomarker for progressive kidney dysfunction (>50% decline in eGFR or ESKD) by multivariable analysis, more so than glomerular staining.

The lectin pathway may be activated when MBL binds to IgM. Like C4d, IgM fluorescence is positive in a variable percentage of IgAN cases, ranging up to 70% (90). Moriyama et al. found IgM deposition to correlate with tuft adhesions but not with renal outcome in Japanese patients (90). Heybeli et al. found IgM to be significantly associated with doubling of serum creatinine or ESKD in Turkish patients (91), and Stefan found such deposition predicted shorter kidney survival in Romanian patients (92). Based on available evidence, the presence of neither C4d nor IgM staining should prompt immunosuppression. More study is needed to determine if therapy directed at the lectin pathway of complement activation should be considered.

## Repeat biopsy in IgAN

The use of repeat biopsy histology as a surrogate endpoint or guide to ongoing immunosuppression has not been adequately studied (see **Table 3**). Two pediatric trials from a single center in Japan were reported (93, 94); one an RCT comparing 2 years of steroids and azathioprine plus standard therapy (heparin–warfarin and dipyridamole) versus standard alone in 86 patients who showed clinical and histologic improvement, including disappearance of mesangial IgA deposits in 7/33 but only with immunosuppression (94). After a mean of 77 months, Hotta et al. rebiopsied 35 adult Japanese patients with initial hematuria and proteinuria that had resolution of hematuria following treatment with warfarin, antiplatelet agents, steroids, and tonsillectomy. They found a significant reduction of mesangial proliferation and disappearance of endocapillary proliferation, necrosis, and cellular crescents, as well as complete loss of IgA deposits in 8 (95). Tumlin et al. found resolution of endocapillary proliferation, cellular crescents, and karyorrhexis after 6 months of steroids and cyclophosphamide in 12 American patients with rapidly progressive, crescentic IgAN that coincided with improved eGFR and reduced proteinuria (96).

**Table 3:** Studies with repeat biopsies.

Author/year	Age	Ethnicity	Number	immunosuppression	Duration between biopsies	Result	Comments
Yoshikawa/1990 (93)	P	Japanese	61	NA	NA	38 Persistent abnormal UA abnormalities 23 Clinical remissions*: Improved histology on LM; reduced mesangial IgA on IF and EDD on EM	Those with persistent UA had progression on LM and no improvement on IF or EM No control group
Yoshikawa/1999 (94)	P	Japanese	78	RCT of SOC (heparin–warfarin and dipyridamole) plus prednisolone/azathioprine for 2 years versus SOC alone	2 years	Reduced proteinuria, reduced glomerular sclerosis, reduced intensity mesangial IgA	Not assessed as a possible surrogate for future outcomes
Hotta/2002 (95)	A	Japanese	35	Prednisolone and tonsillectomy	77 months	Mesangial proliferation significantly reduced Inflammation** in 32 at first biopsy gone	Series with disappearance of hematuria in all 35 and proteinuria in 23 who had a second biopsy
Tumlin/2003 (96)	A	American	12	Pulse steroids and iv cyclophosphamide	6 months	Activity absent on second biopsy	Fared better than historical controls after 36 months
Luo/2014 (97)	A	Chinese	17	Prednisone± Aza ± TwHF 16/17 treated	16 months	IgA and C3 reduced activity decreased chronicity unchanged	Proteinuria reduced from 2.53 to 0.26 g/day
Shen/2015 (98)	A	Chinese	60	Variable: steroids, cyclophosphamide, MMF	10 months	Activity** markedly decreased	Resolution of activity on second biopsy: decreased hematuria/proteinuria
Beckwith/2017 (99)	A	English	18	MMF X 24 months	24 months	Significantly reduced E and C Mesangial IgA significantly reduced	6 Patients had a 3rd biopsy with persistently reduced E

*Complete absence of proteinuria and hematuria.

**Endocapillary proliferation, necrosis, crescents.

A, adult; Aza, azathioprine; C, cellular/fibrocellular crescents per Oxford; E, endothelial hypercellularity per Oxford: EDD, electron-dense deposits; EM, electron microscopy; IF, immunofluorescence; MMF, mycophenolate mofetil; P, pediatric; RCT, randomized controlled trial; SOC, standard of care; TwHF, Tripterygium wilfordii Hook F; UA, urinalysis.

Luo et al. rebiopsied 17 Chinese patients (16 immunosuppressed) after 16 months and found reduced intensity of IgA and C3 staining and reduced activity index without worsening chronicity, which coincided with reduced urinary protein (2.5 g/day to 0.26) (97). Shen et al. rebiopsied 60 immunosuppressed Chinese adults after a mean of 10 months and found significantly reduced endocapillary hypercellularity, crescents, and necrosis coinciding with reduced hematuria/proteinuria only in those with resolution of activity (98). Only interstitial fibrosis/tubular atrophy on the second biopsy was significantly associated with 35% reduction in eGFR or ESKD after a 32-month follow-up. Finally, Beckwith et al. rebiopsied 18 English patients using the Oxford classification after immunosuppression with mycophenolate (99). A significant reduction in the E MEST-C score and cellular/fibrocellular crescents was found along with reduction of mesangial IgA deposition. Notably, after stopping mycophenolate, six patients had additional biopsies that showed persistence of the improved E score.

Although baseline biopsy is an adequate biomarker for prognosis, the currently available data are not sufficient to warrant repeat biopsy as a surrogate endpoint for therapeutic clinical trials. Intuitively, however, ongoing activity suggests the need for active immunosuppression. More study is needed.

## The IgAN risk prediction tool

Barbour et al. derived and externally validated the IgAN Risk Prediction tool by retrospective analysis of 3,927 patients from Europe, North America, China, and Japan which included two prediction models (one with race/ethnicity and one without) with reasonably accurate risk prediction for >50% decline in eGFR or ESKD over a 5-year window (100). Features in the full models included age, sex, race/ethnicity, BMI, renin–angiotensin system (RAS) inhibition (RASi), immunosuppression, eGFR, proteinuria, mean arterial pressure (MAP), the MEST score, and interactions (proteinuria with MAP and with the T score). This tool is available on-line with a mobile app at https://qxmd.com/calculate-by-qxmd. Risk subgroups include low, intermediate, higher, and highest. This has subsequently been retrospectively validated in adult (101, 102) and pediatric cohorts (103). Additionally, since the original analysis evaluated data available around the time of biopsy, the prediction tool was updated to be applied at 1- or 2-year post-biopsy, also available on-line (104). Importantly, the tool has not been validated prospectively nor has it been shown to improve outcomes. The 2021 KDIGO guidelines recommend its use as a guide to inform patients of their risk but not to guide therapy (38).

## Hematuria

The prognostic implication of hematuria depends on the type (macroscopic or microscopic) and the clinical context. Recurrent bouts of gross hematuria have been associated with episodes of AKI. Although initially reported to be benign (105–107) with complete return to baseline kidney function, later studies indicated up to 25% of cases had incomplete recovery with risk factors including age, duration of macroscopic hematuria, red blood cell (RBC) casts, tubular necrosis, and interstitial fibrosis (108). Trujillo published a multicenter series of 26 patients with anticoagulant-related hematuria who developed AKI and found underling biopsy-proven IgAN in 19 of 26 patients with a poor outcome. Only 5 of the 19 had complete recovery and 5 remained dialysis dependent (109).

Whereas gross hematuria is relatively easy to define, the assessment of microscopic hematuria is more problematic and varies between studies. Manual evaluation is often used, with typical cutoffs of 3 or 5 RBCs/high-power field (RBCs/hpf). Microscopic hematuria can be further categorized into lower versus higher levels (110, 111). An automated particle analyzer is available (UF-1000, Sysmex Corporation, Hyogo, Japan), with a proposed cut-off of 28 RBCs/µl. Excellent correlation was found compared to manual evaluation (r = 0.948, RBCs/µl = 5.6 × RBCs/hpf) (112).

Microscopic hematuria is emerging as an important prognostic risk factor, although it was omitted from the International Risk-Prediction Tool in IgAN due to missing data (100). Microscopic hematuria significantly associates with ongoing glomerular inflammation on biopsy (including mesangial hypercellularity, endocapillary proliferation, and crescents) (111). It has been proposed as a signal to initiate immunosuppressive therapy if unremitting and if significant proteinuria coexists (113). In addition to inter-patient variability, there can be significant intra-patient variability over time. Time-averaged hematuria can be used to offset such variability (112, 114–116). Whereas some studies assessed the prognosis of baseline hematuria, others evaluated persistence or remission over time (see **Table 4**).

**Table 4:** Significance of persisting hematuria.

Author/year	Number/ethnicity	Assay	Endpoint	Results	Comments
Sevillano/2017 (117)	112/Spanish	Time-averaged hematuria: Average of 6-month averages of RBCs/hpf Persistence: TAH >5 RBCs/hpf Disappearance: <5 RBCs/hpf in all exams during at least the last 3 years	≥50% decline in eGFR or ESKD Slope of eGFR	Disappearance in 46% Both ESKD and 50% decline significantly lower if no persistence Rate of eGFR decline significantly better with disappearance	Patients with TA proteinuria ≤0.75 g/day had better renal survival Only patients with both TAH >5 and TA-proteinuria >0.75 had worse survival TAH > or TA-proteinuria >0.75 g/day alone no worse than if neither present
Yu/2020 (112)	1,333/Chinese	TAH: mean of 6-month averages Remission (TA): Manual: ≤5 RBCs/hpf Automated: ≤28 RBCs/hpf	≥50% decline in eGFR or ESKD	TAH value-independent risk factor: HR: 1.46 (p = 0.003) Remission of TAH during first 6 months after biopsy: HR: 0.41 (p < 0.001)	Significant interaction of hematuria remission with proteinuria remission (at 1 g/day): Hematuria remission during first 6 months: HR if >1 g/day: 0.46 (p < 0.001) HR if <1 g/day: 0.64 (p = 0.2)
Bobart/2021 (111)	125/American 72 with follow-up	Hematuria definitions: Absent <3 RBCs/hpf Moderate: 3–20 RBCs/hpf Severe: ≥21 RBCs/hpf Present on follow-up: Median ≥3 RBCs/hpf	eGFR change MEST-C scores	Each increase in degree of microhematuria significantly associated with eGFR decline by GEE (p = 0.0001). Baseline hematuria (≥3 RBCs/hpf) associated with M1, E1, C1/2	Baseline proteinuria significantly associated with ESKD Neither baseline hematuria nor isolated follow-up hematuria associated with ESKD
Weng/2022 (116)	152/Chinese	TAH remission: ≤28 RBCs/hpf	Doubling of serum creatinine or ESKD	Significant association with remission: HR: 0.004 (p = 0.010)	Persistent hematuria when associated with both proteinuria ≥1 g/day and low TA-serum albumin had the worst survival
Huang/2023 (110)	684/Chinese (all with persistent hematuria)	228 with the highest tertile persistent hematuria during 6 months after biopsy (≥330 RBCs/µl) propensity matched to 228 with <330 RBCs/µl	≥30% decline in eGFR or ESKD	Composite in 20.2% with highest tertile versus 11.4%, p = 0.02	Greater decrease in eGFR in highest tertile group by GLMM Primary endpoint only significantly worse if crescents were present Both groups had similar proteinuria remission (<1 g/day) Primary endpoint higher in the 684 with persistent hematuria versus 169 with non-persistent hematuria

GEE, generalized estimating equation; GLMM, generalized linear mixed-effects models; TA, time averaged; TAH, time-averaged hematuria.

In IgAN patients with minimal proteinuria, hematuria at presentation is of uncertain significance. Earlier studies of Chinese (118–120), European (121), or Turkish (122) patients found adverse outcomes in some such patients, including increasing proteinuria, hypertension, impaired kidney function, and ESKD. In contrast, more recent studies showed a favorable outlook. Gutierrez et al. evaluated 141 Caucasian patients with microscopic hematuria at baseline, eGFR >60, and proteinuria <0.5 g/day with a 106-month follow-up (123). After 10 years, 96.7% had <50% increase in serum creatinine, as did 99% with <100% increase. After 20 years, the percentages were 91.9% and 99%, respectively. Clinical remission (absence of hematuria, proteinuria <0.2 g/day, normal renal function, normal BP) occurred in 53 (37.5%) by a mean of 60 months with only 12/17 receiving RAS blockers. Proteinuria >0.5 g/day developed in 21.

In Japanese patients with low-level proteinuria (<0.5 g/day), more severe baseline hematuria (>20 RBCs/hpf) fared like those with less severe hematuria regarding increasing proteinuria and kidney survival (124). More severe baseline hematuria did not predict adverse outcomes in Japanese patients even with higher levels of proteinuria (>1 g/day) (125).

He et al. performed a systematic review and meta-analysis of studies regarding the association of hematuria (at disease onset or at biopsy) with adverse outcomes (ESKD, 50% decline in eGFR, or doubling of serum creatinine) and identified 13 studies (5,660 patients) (126). Overall, initial hematuria (gross and microscopic together) was not significantly associated with ESKD (seven studies total), whereas microscopic hematuria considered separately was significantly associated with increased risk (four studies, relative risk 1.21). Macroscopic hematuria was associated with reduced risk (seven studies, RR 0.68). Three studies found no significant association between the magnitude of hematuria and adverse outcomes (111, 124, 125).

Several studies evaluated the significance of persistence or remission of hematuria over time. Le et al. retrospectively analyzed 1,155 Chinese patients with a median follow-up of 5.4 years (114). Time-averaged proteinuria was the strongest predictor of kidney failure, but time-averaged hematuria (defined as the ratio of the area-under-the-curve during follow-up to the duration of follow-up) was also significant by multivariable analysis. For every 10-fold increase, the risk for >50% decline in GFR or ESKD increased by 210%. An optimal cutoff was not established.

Sevillano et al. retrospectively studied 112 Caucasian patients with baseline hematuria for a mean of 14 years and compared those with minimal/negative hematuria (time averaged <5 RBCs/hpf) to those with persistent hematuria (>5 RBCs/hpf); time-averaged proteinuria >0.75 g/day was also compared to <0.75 g/day (117). Multivariable analysis revealed that both time-averaged hematuria and time-averaged proteinuria independently predicted ESKD or 50% eGFR reduction. Notably, 46% had disappearance of hematuria (<5 RBCs/hpf in all urinalyses over final 3 years) with a significant reduction in the rate of eGFR decline. Only patients with both elevated time-average hematuria and proteinuria had significantly worse kidney survival. Neither proteinuria in the absence of hematuria nor the reverse fared worse than having neither.

Using both a manual examination and an automated analyzer, Yu et al. evaluated 1,333 Chinese patients and found by multivariable analysis that time-averaged hematuria (the mean of all 6-month mean levels of hematuria) predicted 50% decline in eGFR or ESKD. Remission of hematuria during the first 6 months (average of <5 RBCs/hpf or <28 RBC/µl) occurred in 19% and significantly predicted a better outcome (112). A significant interaction with proteinuria remission existed such that the benefit of hematuria remission was only seen in those with persistent proteinuria (>1 g/day during the first 6 months) supporting Sevillano’s finding (117) that persisting hematuria is most adverse when coexisting with proteinuria.

Using an automated analyzer, Weng et al. studied 152 Chinese patients and found that persistence of time-averaged hematuria in 96 (defined as area-under-the-curve divided by time of >28 RBCs/µl) independently associated with worse renal outcome (doubling of serum creatinine or ESKD) (116). Receiver operator characteristic curve analysis revealed the optimal cutoff for predicting the adverse outcome was 37 RBCs/µl in males and 201 RBCs/µl in females.

Bobart et al. studied 125 American patients and defined moderate microscopic hematuria as a median of >3–20 RBCs/hpf and severe as >21 RBCs/hpf during follow-up (111). After multivariable adjustment, an increase in the degree of hematuria was significantly associated with decline of eGFR (−0.81, p < 0.011), especially comparing severe hematuria with absent/moderate (−3.99).

Using an automated analyzer, Huang et al. studied 861 Chinese patients and found that 79% had persistent hematuria (median during first 6 months >24 RBCs/µl). After propensity matching, the 228 with high-level hematuria (>330/µl, the upper tertile) to 228 with lower persistent hematuria, they found that the former had a significantly greater risk for the composite end-point (>30% decline in eGFR or ESKD) and a greater decline in eGFR, despite no difference in proteinuria remission (defined as <1 g/day) (110).

Altogether, there remains uncertainty regarding the prognostic significance of baseline hematuria over that provided by other clinical features (proteinuria, BP, eGFR) and the MEST-C score. No data support immunosuppressive therapy based solely on hematuria at baseline. In patients with persistent proteinuria despite 3–6 months of maximal supportive care, the persistence of hematuria indicates ongoing inflammation, portends a worse prognosis, and gives strong support to initiating immunosuppression. Remission of hematuria (repeatedly negative dipstick or <3 RBCs/hpf) despite persistent proteinuria suggests ongoing inflammation may not be present and immunosuppression may have no benefit. Before withholding such therapy, however, a repeat biopsy may be useful. A Japanese Study Group proposed that IgAN remission requires both hematuria remission (<5 RBCs/hpf) and proteinuria remission (<0.3 g/day) be present and persist for 6 months (127). Whereas persisting hematuria may be a potential biomarker, there is no significant evidence that hematuria remission is an adequate surrogate for treatment trials.

## Therapy

As per KDIGO 2021, therapy for IgAN begins with optimizing supportive care. Lifestyle modifications include weight loss, regular exercise, smoking cessation, and sodium restriction. Blood pressure should be controlled with a KDIGO-recommended target of 120 systolic to maximize cardiovascular protection. All patients with proteinuria >0.5 g/day should be on maximally tolerated RASi regardless of blood pressure. At least three RCTs support RASi (128–130). A recent cohort study found proteinuria remission in 55%, including complete remission in 31% (131).

Importantly, KDIGO does not recommend dual RASi with angiotensin-converting enzyme inhibition (ACEi) plus angiotensin receptor antagonist (ARB), as available evidence in IgAN indicates no benefit (132). The potential for harm with ACEi/ARB dual therapy was shown in trials of patients with vascular disease or high-risk diabetes (133, 134). As shown for the nonsteroidal MRA finerenone in diabetic kidney disease (135, 136), whether dual blockade combining either an ACEi or an ARB with a mineralocorticoid antagonist (MRA) (or possibly an aldosterone synthase inhibitor) will benefit IgAN remains to be determined.

Additional antiproteinuric and renal protective therapies include sodium glucose transporter-2 inhibitors (SGLT2i) and endothelin receptor A (ETRA) antagonists (ETRAa). A prespecified analysis of 270 IgAN patients enrolled in the Dapagliflozin and Prevention of Adverse Outcomes in Chronic Kidney Disease Trial revealed a hazard ratio of 0.29 (95% CI 0.12–0.73) for the primary end-point (50% decline in eGFR, ESKD, death from kidney or cardiovascular causes), a 26% reduction in albumin/creatinine ratio, and a lower rate of eGFR decline (137). Similarly, the Study of Heart and Kidney Protection with Empagliflozin (EMPA—KIDNEY) showed a 33% reduction in kidney disease progression (hazard ratio 0.67, 0.46–0.97) in 817 IgAN patients and a 28% reduced slope of decline of eGFR (138). Combining patients from both trials in meta-analysis, kidney disease progression (standardized to ≥50% decline in eGFR, ESKD, or death from kidney disease) was reduced by 51% [relative risk (RR) 0.49, 0.32–0.74] (139). Note that both trials excluded patients with eGFR >90, which does apply to many patients with IgAN.

Endothelin-1 is produced by all renal cell types, interacts with angiotensin II, and via activation of ETRA mediates multiple pathophysiological effects, such as vasoconstriction, inflammation, cell proliferation, and podocyte injury (140). The PROTECT study is an international, double-blind RCT, comparing the dual ETRAa and ARB sparsentan to the ARB irbesartan in 404 IgAN patients with ≥1 g/day of proteinuria, that found a 41% greater protein reduction at 36 weeks with sparsentan leading to accelerated FDA approval (141). After 2-years, proteinuria reduction was maintained (40%), along with significantly greater complete remission (proteinuria <300 mg/day, 31% versus 11%, RR 2.5, 1.6–4.1) and partial remission (<1.0 g/day, RR 1.5, 95% CI 1.1–1.9) (142). The chronic slope (from weeks 6 to 110) of eGFR decline was significantly lower by 1.1/year (p = 0.037), and the total slope (baseline to 110 weeks) was lower by 1.0/year (p = 0.058). The composite endpoint (≥40% reduction in eGFR, ESKD, death) occurred in 9% of sparsentan patients versus 13% irbesartan (RR 0.7, 0.4–1.2). Atrasentan is a selective ETRAa also being studied in IgAN (NCT04573478) (143).

Although not significant in PROTECT, salt and fluid retention are notable side effects of ETRAas. Given the effect of SGLT2is to cause sodium and volume loss, their combination with ETRAas makes intuitive sense. In the SONAR trial of atrasentan in diabetic nephropathy, the 14 patients receiving SGLT2is combined with atrasentan had decreased body weight and a greater decrease in albuminuria compared to 42 propensity match patients receiving only atrasentan (144). In the ZENITH-CKD trial, the ETRAa zibotentan (combined with dapagliflozin) was compared to placebo (plus dapagliflozin) in 449 patients with proteinuric CKD (including 18 IgAN patients) and was found to cause a greater reduction in urinary albumin without significant weight gain (145). The combination of SGLT2i with ETRAa is being evaluated in an extension of PROTECT.

In our opinion, patients with IgAN having either greater than 0.5 g/day proteinuria despite maximally tolerated RASi and eGFR <90 (or eGFR <45 regardless of proteinuria) should receive an SGLT2i. If proteinuria persists despite such dual therapy, an ETRAa should be added.

## Immunosuppression in IgAN

Since IgAN is an immune-mediated disease, the use of immunosuppression is an obvious consideration. The two major questions are *who* to immunosuppress and *how* to. Options include corticosteroids and other anti-inflammatory agents, B-cell inhibition, and anti-complement agents. One size does not fit all, and therapy should be individualized.

As per KDIGO 2021, immunosuppression should only be considered in patients at high risk of progression (proteinuria >0.75–1 g/day) despite maximized supportive care. They do suggest a 6-month course of steroids in high-risk patients. Such patients should be counseled regarding the risks (especially with eGFR <50) and benefits of immunosuppression and offered enrollment in clinical trials. Neither the MEST-C score, the number of crescents, nor the IgAN prediction tool should be used to guide therapy.

## Corticosteroids

Earlier corticosteroid trials targeted proteinuric IgAN but were hampered by a lack of standardized background therapy (RASi) and small sample sizes. Lv et al. performed a systematic review and meta-analysis of trials of steroid therapy for IgAN patients with >1 g/day proteinuria and preserved renal function published through March 2011 and identified nine trials with 532 patients (146). Steroids reduced kidney failure (relative risk 0.32, p = 0.002) and proteinuria (- 0.46 g/day, - 0.63 to – 0.29 g/day) without heterogeneity. By subgroup analysis, high-dose, short-term therapy showed benefit, but low-dose, long-term therapy did not. The quality of studies was low, and all had less than 100 patients. Generalizability was limited.

Subsequently, two large, multicenter, RCTs with conflicting results were published that addressed these concerns, the Supportive Versus Immunosuppressive Therapy for the Treatment of Progressive IgA Nephropathy trial (STOP-IgAN) Trial from Germany and the international Therapeutic Effects of Steroids in IgA Nephropathy Global (TESTING) Trial composed of predominantly (75%) Chinese patients.

In STOP-IgAN, 309 IgAN patients with >0.75 g/day proteinuria completed a 6-month run-in phase of optimized supportive care of which 106 (34%) were excluded due to reduction of proteinuria to <0.75 g/day prior to randomization. Subsequently, 162 were randomized to supportive care only versus immunosuppression with corticosteroids only if eGFR >60 [109 patients, using a regimen showing benefit in a RCT (147)] or corticosteroids plus oral cyclophosphamide for 3 months followed by azathioprine for 33 months if eGFR <60 [53 patients, also derived from a regimen showing benefit in a RCT (148)]. By combining immunosuppression groups, after 3 years, the co-primary endpoints of full clinical remission (protein/creatinine ratio <0.2 with preserved eGFR) was reached by 17% of immunosuppressed patients versus 5% of supportive care only (p = 0.01) and a decrease in eGFR ≥ 15 by 26% and 28%, respectively (p = nonsignificant). Furthermore, there was no significant difference in absolute difference in eGFR decline at 36 months (−4.2 versus −4.7), the slope of the reciprocal of serum creatinine per year, eGFR decline by ≥30, or ESKD between groups. Proteinuria was significantly reduced, but only at 12 months and not at 3 years. Microhematuria (by dipstick), present in 87% initially, remitted in 42% with immunosuppression versus 16% supportive only (p = 0.004).

A *post-hoc* analysis of STOP-IgAN considered the two immunosuppression protocols and their respective controls separately. The steroid-only group (eGFR ≥60) had greater full clinical remission but no difference in eGFR loss ≥15 versus its respective control group. Immunosuppression in the lower eGFR group had a significant difference in neither full remission nor eGFR decline ≥15 versus its respective control group (149). Severe infections, impaired glucose tolerance, and weight gain were significantly more common with immunosuppression.

In long-term follow-up of 149 patients of STOP-IgAN (median 7.4 years), the primary endpoint (>40% decline in eGFR, ESKD, total mortality) occurred in 45.5% with immunosuppression versus 50% with supportive care, a non-significant difference, with no significant differences when both immunosuppression regimens were compared with respective controls (150). Neither annual eGFR loss nor rate of eGFR loss >40% differed with immunosuppression.

In contrast, 503 patients (5% Caucasian/European) in TESTING were randomized to oral methylprednisolone (initially 0.6–0.8 mg/kg/day, maximum 48 mg/day, with a reduced dose protocol of 0.4 mg/kg, maximum 32 mg/day due to excessive infections with the higher dose) or placebo for 6–9 months with a 3.5-year follow-up. The primary endpoint (eGFR decline ≥40%, ESKD, or death due to kidney failure) was significantly reduced with steroids [hazard ratio (HR) 0.53, 0.39–0.72] with similar effects with both dosages. The rate of eGFR decline was significantly slower with steroids (−2.46/year, p = 0.002), as was development of ESKD (HR 0.59, p = 0.008). Time-averaged proteinuria was reduced by 0.69 g/day but, like STOP-IgAN, was no longer significantly different at 36 months. Serious adverse events (mostly hospitalizations and serious infections) were more frequent with steroids, especially the higher dose.

Since a major source of Gd-IgA1 production is gut-associated lymphoid tissue (Peyer’s patches) in the terminal ileum, a targeted-release formulation of the oral steroid budesonide (Nefecon) was developed that despite direct targeting of Peyer’s patches has first-pass hepatic clearance and less systemic steroid exposure. Nefecon demonstrated significant proteinuria reduction in a phase 2b trial (NEFIGAN) (151). Subsequently, part A of the phase 3 NefIgArd trial, a multicenter, double-blind trial of Nefecon 16 mg/day for 9 months, showed a 27% lower degree of proteinuria at 9 months versus placebo with better eGFR (3.78 difference).

The final results of NefIgArd included 364 primarily Caucasian patients with proteinuria >1 g/day or urine protein/creatinine ratio ≥0.8 g/g and eGFR 35–90 despite 3 months of RASi randomized to 16 mg/day Nefecon or placebo for 9 months with a 2-year follow-up. The primary endpoint, time-weighted average eGFR over 2 years, showed a significant benefit of 5.05 (p < 0.0001) with a 2-year total slope difference of 2.95/year favoring Nefecon (representing 50% less deterioration) and with no interactions based on baseline proteinuria. Proteinuria was reduced by 40% over months 12 to 24, but the maximal effect was at 12 months (~50%) that decreased by 24 months (~30%). Steroid-related side effects included edema, hypertension, muscle spasm, and headache. The risk of infection was no greater.

Overall, the best available data indicate that steroids are beneficial, especially in Chinese patients, but their effect is most pronounced while being given and appears to wane after discontinuation. It remains uncertain to what extent a relatively short course of therapy will affect the long-term progression of a disease evolving over decades in most patients. The dosages and therapy durations shown to be effective produce significant toxicity. Hence, case-by-case consideration is required. In our opinion, those demonstrating significant eGFR decline, persisting proteinuria, and hematuria while on maximal supportive therapy would gain the most benefit. Nefecon appears effective with less toxicity than prednisone but is much more expensive.

## Targeting B-lineage cells

Central to the pathogenesis of IgAN is overproduction of Gd-IgA1 from B lymphocytes and plasma cells, the latter residing predominantly in mucosal-associated lymphoid tissues (MALT) of the gastrointestinal or respiratory tracts (156). Normal maturation of B-lineage cells to plasma cells, including class switching to IgA (157), can occur independent of T cells through interactions of the B-lineage survival factors B-cell-activating factor (BAFF) and a proliferation-induced ligand (APRIL) produced by dendritic cells and monocytes/macrophages in the lamina propria (157, 158). BAFF interacts with immature B cells through the BAFF receptor (BAFF-R), but also at later stages through interaction with transmembrane activator and calcium modulator and cyclophilin ligand interactor (TACI) present on both mature naïve B cells and plasma cells and B-cell maturation antigen (BCMA) present on plasma cells (158). APRIL only interacts with TACI and BCMA, not BAFF-R, and is most important at later stages, i.e., memory B cells and plasma cells (158).

Normal B-cell maturation and class switching to IgA also occur in a T-cell-dependent manner in secondary lymphoid organ germinal centers (e.g., Peyer’s patches) through interaction with antigen-primed T cells (T-follicular/helper cells), with synergy from BAFF and APRIL (157). Mature IgA-secreting plasma cells normally home to survival niches in MALT but may also mishome to the bone marrow.

By targeting B cells, Lafayette et al. randomized 34 proteinuric IgAN patients to rituximab plus standard of care versus standard of care alone and found no difference after 1 year in proteinuria reduction or eGFR (**Table 5**). Serum levels of both Gd-IgA1 and anti-Gd-IgA1 did not change despite peripheral B-cell depletion, supporting a primary role of plasma cells in the production of these pathogenic antibodies (152). Whether more potent, second-generation, anti-CD20 monoclonal antibodies (ofatumumab or obinutzumab) will be more effective in IgAN remains to be determined. The utility of biologic agents targeting the later stages of B-cell development (i.e., plasma cells), such as the anti-CD38 monoclonal antibodies daratumumab and felzartamab (NCT05065970) remains to be determined. Proteosome inhibition with bortezomib is also under investigation (NCT01103778).

**Table 5:** Targeting B-lineage cells in IgAN.

Class	Agent	Administration	Published trials	Results	Comments	Pending trials
Anti-CD20 antibodies	Rituximab	Intravenous	Lafayette/2017 (152)	Successful peripheral B-cell depletion No benefit on eGFR, proteinuria, Gd-IgA1, or anti-Gd-IgA1 antibody levels	Suggest plasma cells as main source of Gd-IgA1	NCT02571842 NCT05824390 NCT02571842
Anti-APRIL antibodies	Sibeprenlimab	Intravenous	Mathur/2023 (153)	Reduced 24-h proteinuria	Await phase 3 trials Appears safe	NCT05248646 NCT05248659
Combined BAFF/APRIL inhibition	Telitacicept	Intravenous	Lv/2023 (154)	Reduced proteinuria eGFR stable	Await phase 3 trials Appears safe	NCT05596708 NCT05799287 NCT04905212
	Atacicept	Intravenous	Barratt/2022 (155)	Reduced proteinuria Reduced Gd-IgA1 levels	Await phase 3 trials Appears safe	NCT04716231
	Povetacicept	Intravenous	NA	NA	Not restricted to IgAN patients	NCT05732402

APRIL, a proliferation-induced ligand; BAFF, B-cell-activating factor; Gd-IgA1, galactose-deficient IgA1; NA, not available.

Neutralizing BAFF and/or APRIL is germane to the pathophysiology of IgAN given their role in both T-cell-dependent and -independent class switching of B cells to produce IgA (**Table 5**). BAFF is involved in the earlier stages of B-cell maturation, and APRIL is involved in the later stages of B-cell maturation and plasma cell survival (158). Evidence indicates that both BAFF (159) and APRIL (160) are involved in experimental IgAN. In IgAN serum levels of BAFF are elevated and correlate with clinical and pathologic features (161). APRIL levels are also elevated and correlate with levels of Gd-IgA1 (162), as well as with progression to ESKD (163).

Multiple agents targeting BAFF and/or APRIL are under investigation (152–155). Blisibimod is a hybrid protein made in *Escherichia coli* targeting both membrane-bound and soluble BAFF. BRIGHT-SC (NCT02062684) was a study of subcutaneous blisibimod therapy in IgAN patients that had favorable results presented orally in 2016 but never published. A phase 3 trial was withdrawn.

Sibeprenlimab, a humanized IgG2-monoclonal antibody that neutralizes APRIL, was tested in a phase 2 trial (ENVISION) of 155 predominantly (~75%) Asian IgAN patients given sibeprenlimab 2, 4, 8 mg, or placebo subcutaneously monthly for 12 months (153). The geometric mean ratio reduction from baseline in 24-h urine protein/creatinine ratio was 49.6%, 56.7%, 62.8%, and 12.5%, respectively (p < 0.001 for linear trend). Clinical remission (<300 mg/day proteinuria) at 12 months occurred in 7.9%, 12.2%, 26.3%, and 2.6%, respectively. The slope of decline of eGFR was reduced in all three sibeprenlimab groups versus placebo. Serum levels of APRIL, IgA, IgM, IgG (less so), and Gd-IGA1 were suppressed during the 12 months but rebounded after 4 months. There were no safety concerns. A phase 3 trial is ongoing (VISIONARY, NCT05248646). Zigakibart (BION-1301) is a monoclonal antibody also targeting APRIL that showed efficacy (presented in 2021 but not published). A phase 3 trial is underway (BEYOND, NCT05852938).

Fusion proteins containing the TACI receptor inhibit both BAFF and APRIL. Telitacicept, a recombinant fusion protein of TACI and the Fc component of human IgG that binds and neutralizes both BAFF and APRIL, was studied for the treatment of lupus nephritis (164, 165). Lv et al. reported a phase 2 trial in 44 proteinuric Chinese IgAN patients given telitacicept 240 mg, telitacicept 160 mg, or placebo weekly for 24 weeks and found a 0.889-g/day (49%) reduction in mean proteinuria with 240 mg/week (least squared mean difference versus placebo −0.88, p = 0.013) but a lesser and nonsignificant reduction with 160 mg/week; eGFR remained stable in both telitacicept groups but fell significantly with placebo (154). IgA, IgG, and IgM levels decreased only with telitacicept.

Atacicept, also a human recombinant fusion protein of IgG1 and TACI under investigation for autoimmune diseases [systemic lupus (166)], was studied in the small phase 2a JANUS trial, comparing once weekly injections of atacicept 25 mg, atacicept 75 mg, and placebo in 16 proteinuric IgAN patients that was terminated early due to slow enrollment (155). No safety signals emerged, and immunoglobulin levels, including Gd-IgA1, were reduced. Early proteinuria reduction occurred that was maintained only with the 25-mg dose; eGFR was stable with atacicept but declined with placebo. The phase 2b ORIGIN trial (NCT04716231) randomized 116 patients with proteinuric IgAN to 150 mg of atacicept, 75 mg of atacicept, or placebo weekly for 36 weeks and found significantly greater reduction in proteinuria versus placebo at 24 weeks (25% combining both atacicept arms, p = 0.037), stability of eGFR, and a 60% reduction in Gd-IgA1 (167). A phase 3 trial comparing the 150-mg dose with placebo (ORIGIN 3) is ongoing. A third fusion protein dual BAFF/APRIL inhibitor, povitacicept, is under study for glomerulonephritis, including IgAN (NCT05732402).

Overall, APRIL and/or BAFF inhibition appear to be a reasonably well-tolerated approach to reducing the pathophysiologic driver of IgAN (Gd-IgA1 and anti-Gd-IgA1 antibodies). However, like steroids, the effect is likely to persist only while on therapy. Phase 3 trials demonstrating effectiveness and safety are required before recommendations can be made.

## Complement inhibition in IgAN

The complement system is intricately involved in the pathogenesis of IgAN given the correlation of disease severity with both serum markers of activation and tissue deposition of components. Polymorphisms of complement-regulating genes are also linked to susceptibility to IgAN. The primary pathway of complement activation in IgAN is the alternate pathway. The lectin pathway may be involved in a significant minority of patients and appears to correlate with disease severity. Despite the frequent co-deposition of IgG and/or IgM, the classic pathway is apparently not involved as C1q is rarely found. The variability in complement activity and regulation from patient to patient allows for potential personalized therapeutic choices given the diversity of drugs currently under investigation and the pathways they target (**Table 6**) (168–172).

**Table 6:** Complement inhibition in IgAN.

Target	Agent	Administration	Published trials	Results	Comment	Pending trials
Factor B	Iptacopan	Oral	Zhang/2024 (168)	24% Reduction in 24-h urine P/C ratio at 3 months	Further decrease in proteinuria at 6 months Appears safe Phase 3 trial awaited	NCT04557462 NCT04578834
	IONIS-FB-L_RX_	Subcutaneous	NA	NA	NA	NCT04014335
Factor D	Vermircopan	Oral	NA	NA	NA	NCT05097989
	Pelecopan	Oral	NA	NA	NA	NCT05162066
MASP-2	Narsoplimab	Intravenous	Lafayette/2020 (169)	54%–95% Reduction of 24-h UPE at 18 weeks in patients on tapering steroids No benefit in RCT of steroid naïve at 18 weeks	Phase 3 trial terminated by sponsor for lack of efficacy	NCT02682407
C3	Pegcetacoplan	Subcutaneous	Dixon/2023 (170)	1.1% Reduction in 24-h urine P/C ratio at 48 weeks	Only 6 IgAN patients Included Ce glomerulopathy and lupus patients	NA
C5	Cemdisiran	Subcutaneous	Barratt/2024 (171)	37.4% Reduction in 24-h urine P/C ratio at 32 weeks 98.7% reduction in serum C5 at 32 weeks	Well-tolerated Phase 2	NA
	Ravulizumab	Intravenous	NA	NA	NA	NCT06291376
C5 receptor	Avacopan	Oral	Bruchfeld/2022 (172)	6 of 7 Patients had improve slope of urine P/C ratio	Small and uncontrolled	NA

MASP-2, mannan-binding lectin associated serine protease-2; NA, not available; P/C, protein/creatinine; UPE, urine protein excretion.

Several inhibitors of the alternate pathway are being studied. A double-blind placebo-controlled phase 2 dose-finding study (10, 50, 100, or 200 mg bid) of the oral Factor B inhibitor iptacopan found a significant 23% reduction in 24-h urinary protein/creatinine ratio with the 200-mg bid dose at 3 months with further reduction at 6 months. Evidence of alternate pathway inhibition included significant biomarker reductions (plasma Bb levels, serum *ex vivo* alternate pathway activity by the Wieslab assay, and urinary C5b-9 levels) (168). A phase 3 trial of iptacopan in IgAN is ongoing (APPLAUSE-IgAN, NCT04578834). IONIS-FB-L_Rx_, an antisense inhibitor of Factor B administered by subcutaneous injection to IgAN patients, had positive results to reduce urinary protein by 44% at week 29 as per company press release (Ionis Pharmaceuticals) in an unpublished phase 2 study. A phase 3 study is active (NCT04014335). Two oral Factor D inhibitors, pelecopan and vemircopan, are being studied for IgAN, but published results are not available.

Involvement of the lectin pathway of complement activation is based on glomerular staining of MBL, ficolins, and C4d in the absence of C1q. A humanized antibody targeting the effector enzyme MASP-2, narsoplimab, was found in a phase 2 study of 16 patients to be safe and well tolerated and reduced proteinuria up to 95% in patients on steroids and up to 61% in those not on steroids (169). However, the phase 3 trial of narsoplimab, ARTEMIS-IGAN (NCT03608033), was terminated by the sponsor, due to lack of efficacy in reducing proteinuria.

The common pathway has been targeted with multiple agents. Cleavage of C3 results in the anaphylatoxin C3a and C3b, the latter required for both amplifying all three pathways and completing their respective C5-convertases. Pegcetacoplan, extensively studied and approved for paroxysmal nocturnal hemaglobinuria (173), binds to C3 preventing its cleavage without interfering with the tickover pathway (similar to compstatin) (174). In a phase-2, 48-week, basket study in 21 patients with glomerulonephritis, including 6 with IgAN, pegcetacoplan was found to have a favorable safety profile, although proteinuria was only reduced from 1.3 g/day at baseline to 1.2 g/day at 48 weeks in IgAN patients (170).

Cemdisiran, a subcutaneous small interfering RNA therapeutic that inhibits C5 production, was studied in a 36-week, placebo-controlled, phase 2 trial of 31 IgAN patients. Findings included a placebo-adjusted 37.4% reduction of mean 24-h urine protein/creatinine ratio with serum C5 reduction of 98.7% (versus 25% with placebo), reduced dipstick-grade hematuria, and less decline of eGFR than placebo (171). Serum complement alternate pathway activity was reduced by 48% and classic pathway activity was reduced by 77% versus little change with placebo.

Eculizumab is a humanized monoclonal antibody targeting C5 approved for atypical HUS, which has shown transient benefit in case reports of aggressive IgAN (175, 176). Raviluzumab, another humanized anti-C5 monoclonal antibody, is being studied in a placebo-controlled trial in 450 projected IgAN patients (NCT06291376). Avacopan is an oral C5a receptor inhibitor approved for use in ANCA-vasculitis (177). In an open-label pilot study of seven IgAN patients, avacopan 30 mg bid for 12 weeks produced a 50% improvement in proteinuria in three patients (172). Terminal complement inhibition would seem to be most beneficial when TMA and/or strong glomerular C5b-9 staining are found on biopsy, but this remains to be determined.

Complement inhibition holds promise for the treatment of IgAN, but like BAFF and APRIL, phase 3 studies are required to demonstrate effectiveness and safety, and to better determine which patients would most likely respond to which agents. Given the effectiveness of complement inhibition in atypical hemolytic–uremic syndrome, it remains to be determined if the same applies to IgAN patients showing TMA on biopsy.

## Other immunosuppressants in IgAN

Cyclophosphamide has been used together with steroids for clinically severe IgAN especially associated with necrosis, inflammation, and/or crescents. Nonrandomized studies have used oral (178, 179) or intravenous (96, 180) cyclophosphamide and compared outcomes to historical controls or contemporary untreated patients. Standardized definitions of either clinical course or pathologic severity were unavailable for inclusion into these studies for appropriate comparisons. Ballardie and Roberts performed an RCT in 38 patients with rapidly progressive primary IgAN defined as creatinine >130 µmol/L rising by ≥15% in the prior year with projected ESKD within 5 years. They compared no immunosuppression to tapering oral steroids plus oral cyclophosphamide 1.5 mg/kg/day for 3 months followed by oral azathioprine 1.5 mg/kg for up to 2 years (the regimen used in STOP-IgAN) and showed a reduced rate of renal function decline that was arrested in one-third with reductions in proteinuria and erythrocyturia (148). Per KDIGO, patients with a rapidly progressive course, defined as ≥50% decline in eGFR in ≤3 months in the absence of other causes with a biopsy typically showing a high percentage of crescents and focal necrosis, should be offered cyclophosphamide and steroids (38).

Multiple observational studies and RCTs assessed mycophenolate mofetil (MMF) in IgAN. Earlier meta-analyses (2008–2009) of RCTs found no significant benefit (181–183). Chen et al. performed a 2014 meta-analysis identifying eight RCTs (357 patients) and found no significant effects on proteinuria or renal function; however, studies employing short-term therapy had a benefit (184). Three of the eight trials (69 patients) found MMF superior to cyclophosphamide. A 2021 meta-analysis of nine trials (587 patients) comparing MMF ± steroids to placebo or full-dose steroids found no benefit on remission, proteinuria reduction, doubling of serum creatinine, or ERKD. Cushing’s syndrome was less, but infection was more with MMF compared to full-dose steroids (185).

The Effect of Mycophenolate Mofetil on Renal Outcomes in Advanced Immunoglobulin A Nephropathy trial (MAIN) was an RCT of MMF plus standard-of-care versus standard-of-care alone for 3 years in 170 Chinese IgAN patients with proteinuria >1 g/day. Treatment with MMF significantly reduced the two primary outcomes of a composite endpoint (doubling of serum creatinine, ESKD, renal or cardiovascular death, HR 0.23, 0.09–0.63) and progression of kidney disease (≥30% decline of eGFR from >60 to <60 or ≥50% decline if baseline eGFR <60, adjusted HR 0.23, 0.10–0.57) (186). In those patients stopping MMF at trial end, however, the eGFR decline during post-trial follow-up increased from −2.9/year during the trial to −6.1/year afterward. Based on these results, patients at high risk for both steroid side effects and progression can be considered for MMF, especially if Chinese.

Leflunomide, a dihydroorotate dehydrogenase inhibitor that suppresses lymphocyte proliferation, has been studied in multiple small trials. A systematic review and meta-analysis evaluated 44 studies of 1,802 patients (35 RCTs) and found both lower proteinuria and serum creatinine with leflunomide combined with steroids versus steroids alone (27 studies, 1,802 patients); 6 studies comparing leflunomide with RASi versus RASi alone (664 patients) also found significantly lower proteinuria and serum creatinine (187). A more recent meta-analysis restricted to low-dose steroids plus leflunomide versus full-dose steroids included three trials (342 patients) and found reduced urine protein with the combination, although there was no significant difference in renal function (188). No safety signals emerged from either meta-analysis.

Hydroxychloroquine, now standard of care for lupus nephritis, may also be effective in IgAN by inhibiting Toll-like receptor-9 upregulation of BAFF/APRIL and cytokine production, as well as by inhibiting antigen processing by antigen-presenting cells. A 2021 systematic review uncovered one RCT (60 patients), one prospective case-control study (28 patients), two retrospective case-control studies (236 patients), and one observational cohort study (180 patients) all restricted to Chinese patients (189). In the RCT, hydroxychloroquine plus losartan significantly reduced proteinuria versus losartan alone. The other studies supported proteinuria reduction overall. No effect on eGFR was detected.

He et al. performed a retrospective cohort study of 159 Chinese IgAN patients with moderate proteinuria (~0.8 g/day) given hydroxychloroquine + RASi, leflunomide + RASi, or RASi alone followed for 6 months (190). Compared with RASi alone, both active treatment groups had significantly lower proteinuria, with hydroxychloroquine having a significantly greater effect than leflunomide. Conversely, only leflunomide significantly reduced hematuria compared to either RASi alone or hydroxychloroquine. Overall, the data are insufficient to recommend either hydroxychloroquine or leflunomide for routine treatment of IgAN.

## Tonsillectomy

Mucosal-associated lymphoid tissues, including Peyers patches and Waldeyer’s ring, are considered a likely source of Gd-IgA1 and associated antibodies (156). The clinical occurrence of gross hematuria concurrently or shortly following an upper respiratory infection (synpharyngitic nephritis) supports a prominent role of oral pharyngeal lymphoid tissue. By studying tonsillar plasma cells of IgAN patients, Bene et al. found a predominance of IgA- versus IgG-plasma cells, a reversal of the findings in patients with recurrent tonsillitis (191).

Tonsillectomy has received considerable attention for the treatment of IgAN (192). First reported from a Germen study in 1999 (193), tonsillectomy has gained wide acceptance in Japan. A 2011 meta-analysis reviewed 7 retrospective trials (858 patients, 6 from Japan, 1 from China) and found that remission was higher, but only when combined with pulse steroids, and not when used alone (194). A 2015 meta-analysis reviewed 14 mostly retrospective studies (1,794 patients), 12 from Japan and 1 from China, and found significantly greater odds of remission with tonsillectomy [10 studies, 1,431 patients, odds ratio (OR) 3.40, p < 0.001] and decreased odds of ESKD (9 studies, 873 patients, OR 0.25, p < 0.001) (195). A meta-analyzed 19 studies (15 from Japan, 2 from China) with 3,483 patients found a higher rate of remission (15 studies, 3,059 patients, OR 3.30, 2.47–4.40) and reduced ESKD (9 studies, 1,804 patients, OR 0.33, 0.16–0.69) with tonsillectomy (196). Most studies were observational and mostly retrospective.

There have been two RCTs of tonsillectomy. Kawamura and colleagues randomized 72 Japanese patients to steroid pulses with or without tonsillectomy and found significantly greater chance of proteinuria remission (OR 2.98, p = 0.049) but neither hematuria remission nor combined hematuria/proteinuria remission with tonsillectomy (197). On subsequent analysis, patients having tonsillectomy were more likely to have both proteinuria remission and combined remission versus no tonsillectomy, only if there were more advanced histological changes (198). Yang et al. randomized 98 Chinese IgAN patients to tonsillectomy plus drug therapy to drug therapy alone (the latter consisting of tapering *Tripterygium* glycosides in all patients and tapering prednisone if proteinuria >1 g/day) and found a significantly shorter time to proteinuria remission (2.5 versus 26.1 months, p < 0.001), hematuria remission (3.1 versus 24.9 months, p <0.001), greater cumulative remission rates for both hematuria and proteinuria, duration of remissions, and lower relapse rates with tonsillectomy (199).

Feehally et al. evaluated 1,147 European IgAN patients and found that 41 had tonsillectomy at some point for tonsillitis or obstructive sleep apnea, including 17 with the procedure performed after IgAN diagnosis (200). Comparing the 41 to 41 propensity-matched controls, there was no difference in either the rate of eGFR decline (−1.9/year versus −0.5/year, p = 0.105) or in the composite of ≥50% reduction in eGFR or ESKD (3/41 versus 8/41, p = 0.105); the same result obtained comparing the 17 with post-diagnosis tonsillectomy to 51 propensity-matched controls. These data do not support tonsillectomy for Caucasian patients, but tonsillectomy deserves consideration in Japanese patients.

## Discussion

Primary IgAN displays variable clinical manifestations at presentation that progresses to ESKD in up to 50% of patients. Those of Pacific-Asian origin are at greater risk and those of African origin less so compared to Caucasians. There are no non-invasive biomarkers for diagnosis capable of replacing biopsy, which remains the gold standard. The Oxford classification should be used to standardize interpretation. Future iterations of the Oxford classification hopefully will address the significance of TMA, C4d staining, and podocyte abnormalities, including possible subclassification of S1.

The best available biomarkers for predicting progression at the time of presentation or initial biopsy include proteinuria, reduced eGFR, hypertension, and MEST-C score. Time-averaged proteinuria and slope of eGFR decline can further assess risk over time. Time-averaged hematuria requires more study. Other potential serum and urine biomarkers require verification. The IgAN Prediction tool can be used to better inform patients of their risk.

The backbone of treatment applicable to all patients is lifestyle modification, including sodium restriction, exercise, weight loss, smoking cessation, and strict control of blood pressure (<130/80 as a minimum, consider <120/70 as per KDIGO). RASi should be given to all patients with proteinuria (≥300 mg/g creatinine or ≥500 mg/day) regardless of blood pressure. SGLT2i are indicated for persisting albuminuria (albumin/creatinine ratio >200) or eGFR <45 regardless of albuminuria (based on inclusion criteria of EMPA—KIDNEY). ETRAa should be considered if proteinuria persists.

The use of immunosuppression should be individualized and considered after an adequate trial of supportive therapy (3–6 months). Exceptions requiring immediate therapy include nephrotic syndrome from MCD–IgAN, which should be treated with high-dose steroids as adult MCD, and those having a rapidly progressive course with necrosis and crescents on biopsy, where cyclophosphamide and steroids are recommended.

Otherwise, the most obvious case requiring immunosuppression is a younger patient with declining eGFR and persisting proteinuria despite maximal supportive therapy, especially with persisting hematuria as an indicator of ongoing inflammation. KDIGO suggests persisting proteinuria >0.75–1 g/day as the main indicator for high risk, but as shown in RaDaR, patients with <500 mg/day may still progress. Hence, all factors must be considered, especially declining eGFR and persisting hematuria even with lower levels of proteinuria.

Older patients with comorbidities capable of contributing to declining GFR and proteinuria (such as diabetes, hypertension, and vascular disease) favor not treating especially given the higher risk of immunosuppression in the elderly. Asian patients have a more aggressive course, and Chinese patients have shown significant responses to immunosuppression (steroids in TESTING and MMF in MAIN) supporting a more aggressive course in such patients. Further support for use of steroids and/or MMF in Chinese patients comes from a large observational database of 3,946 patients from which new users of immunosuppression were propensity-matched to patients treated only with supportive care that found a 40% reduction in hard endpoints with similar effect sizes with glucocorticoids alone, MMF alone, or both (201).

If a significant time has elapsed since diagnostic biopsy and hematuria is not persisting, rebiopsy may be indicated to determine if ongoing proteinuria is the result of chronic, irreversible changes (glomerular sclerosis and interstitial fibrosis) and not ongoing inflammation, in which case therapy would not be indicated.

The main alternatives include a 6-month course of high-dose oral steroids (with or without iv boluses) versus targeted-release budesonide. The latter reduces systemic steroid exposure but comes at significantly greater cost. Antibiotic prophylaxis and skeletal protection are required with oral steroids. Alternatively, in patients at higher risk of steroid toxicity, MMF can be considered, especially if Chinese. Tonsillectomy may be considered in Japanese patients, probably combined with bolus steroids. The roles of leflunomide and hydroxychloroquine remain uncertain.

Whereas such nonspecific immunosuppression may effectively quell inflammation while being administered, the underlying genetic tendency and pathophysiology are unlikely to change, i.e., enhanced Gd-IgA1 production and polymerization together with the enhanced production of anti-Gd-IgA1 antibodies, immune complex formation, mesangial deposition, complement activation, and mesangial cell activation/proliferation. High-dose immunosuppression cannot be continued for prolonged periods given obvious toxicity risks.

Multiple agents are being studied to offer a more targeted approach. Targeting APRIL alone or BAFF plus APRIL may reduce production of Gd-IgA1 by inhibiting class switching of developing B cells and reducing plasma cell survival. Phase 2 studies are promising, and phase 3 studies are awaited. It remains to be determined if the risk benefit ratio favors targeting APRIL alone or both BAFF and APRIL. Given the obvious role of complement, especially the alternate pathway but also the lectin pathway, multiple steps are potential targets with multiple agents under investigation in phase 2 and 3 studies. These include Factor B and D inhibitors, MMASP inhibitors, and C3 and C5 inhibitors. The best way to suppress complement in IgAN is uncertain and may vary from patient to patient. Perhaps serum, urine, and/or tissue biomarkers can help determine the best choice for an individual patient.

Ultimately, we suspect that combinations of agents will give the best results. It remains to be determined if they should be applied concurrently or sequentially and if multiple courses of an agent found to be effective in a given patient should be used. IgAN remains a fascinating disease under active study. Hopefully, IgAN can be relegated to a very uncommon cause of ESKD and shortened life expectancy.

## Author contributions

EF: Conceptualization, Data curation, Writing – original draft, Writing – review & editing. RG: Writing – review & editing. JF: Writing – review & editing.
